# Pepper immunity against *Ralstonia solanacearum* is positively regulated by *CaWRKY3* through modulation of different WRKY transcription factors

**DOI:** 10.1186/s12870-024-05143-z

**Published:** 2024-06-10

**Authors:** Ansar Hussain, Abdul Qayyum, Shahid Farooq, Saeedah Musaed Almutairi, Rabab Ahmed Rasheed, Masood Qadir, Tomáš Vyhnánek, Yunhao Sun

**Affiliations:** 1 Innovative Institute for Plant Health, Zhongkai University of Agriculture and Engineering, Guangzhou, 510225 China; 2 Department of Plant Breeding and Genetics, Ghazi University, Dera Ghazi Khan, 32200 Pakistan; 3 Department of Plant Breeding and Genetics, Faculty of Agricultural Science and Technology, Bahauddin Zakariya University, Multan, 60800 Pakistan; 4 Department of Plant Protection, Faculty of Agriculture, Harran University, Şanlıurfa, 63050 Türkiye; 5 Department of Botany and Microbiology, College of Science, King Saud University, P.O. 2455, Riyadh, 11451 Saudi Arabia; 6 Histology & Cell Biology Department, Faculty of Medicine, King Salman International University, South Sinai, Egypt; 7 Department of Plant Biology, Faculty of AgriSciences, Mendel University in Brno, Zemedelska 1, Brno, 61300 Czech Republic

**Keywords:** *CaWRKY3*, *Capsicum annum*, Transcription factor, Immunity, *Ralstonia solanacearum*

## Abstract

**Background:**

Several WRKY transcription factors (TFs), including *CaWRKY6*, *CaWRKY22*, *CaWRKY27*, and *CaWRKY40* are known to govern the resistance of pepper (*Capsicum annuum* L.) plants to *Ralstonia solanacearum* infestation (RSI) and other abiotic stresses. However, the molecular mechanisms underlying these processes remain elusive.

**Methods:**

This study functionally described *CaWRKY3* for its role in pepper immunity against RSI. The roles of phytohormones in mediating the expression levels of *CaWRKY3* were investigated by subjecting pepper plants to 1 mM salicylic acid (SA), 100 µM methyl jasmonate (MeJA), and 100 µM ethylene (ETH) at 4-leaf stage. A virus-induced gene silencing (VIGS) approach based on the Tobacco Rattle Virus (TRV) was used to silence *CaWRKY3* in pepper, and transiently over-expressed to infer its role against RSI.

**Results:**

Phytohormones and RSI increased *CaWRKY3* transcription. The transcriptions of defense-associated marker genes, including *CaNPR1*, *CaPR1*, *CaDEF1*, and *CaHIR1* were decreased in VIGS experiment, which made pepper less resistant to RSI. Significant hypersensitive (HR)-like cell death, H_2_O_2_ buildup, and transcriptional up-regulation of immunological marker genes were noticed in pepper when *CaWRKY3* was transiently overexpressed. Transcriptional activity of *CaWRKY3* was increased with overexpression of *CaWRKY6*, *CaWRKY22*, *CaWRKY27*, and *CaWRKY40*, and vice versa. In contrast, *Pseudomonas syringae* pv tomato DC3000 (Pst DC3000) was easily repelled by the innate immune system of transgenic *Arabidopsis thaliana* that overexpressed *CaWRKY3*. The transcriptions of defense-related marker genes like *AtPR1*, *AtPR2*, and *AtNPR1* were increased in *CaWRKY3*-overexpressing transgenic *A. thaliana* plants.

**Conclusion:**

It is concluded that *CaWRKY3* favorably regulates phytohormone-mediated synergistic signaling, which controls cell death in plant and immunity of pepper plant against bacterial infections.

**Supplementary Information:**

The online version contains supplementary material available at 10.1186/s12870-024-05143-z.

## Background

Plants (being immobile) are vulnerable to several abiotic stresses, disease and insect infestations, and harsh environments, including high temperatures, water scarcity, and excessive salinity [1, 2]. Plants overcome these challenges by using coordinated responses, either through initiation of transcriptional reprogramming or regulation of diverse transcription factors (TFs). Nevertheless, the underlying mechanism responsible for this synchronization remains unspecified.

Plants use dual immunity to protect themselves against disease infestations [3, 4]. Pathogen associated molecular pattern (PAMP)-triggered immunity (PTI) (often referred as basal defense) is initiated by the identification of PAMPs on the cell surface by trans-membrane receptors (PRRs) (pattern recognition receptors). Effector-triggered immunity (ETI) is the second layer, and activated in response to pathogen-made effectors that are released into the host cell and work to dampen PTI by destroying different PTI-signaling components [5]. The ETI is severe, protracted, and coupled with localized cell death at infection site and systemic acquired resistance in most instances [6]. Despite these differences, PTI and ETI have several comparable signaling processes such as Ca^+ 2^ signaling, oxidative burst, activation of mitogen-activated protein kinase (MAPKs), and transcriptional control of defense-related genes through several TFs [6, 7]. A few of these elements have been connected to the way plants react to various stressful conditions. This raises the possibility that these elements have a regulatory function in regulating how plants react to diverse stress stimuli. Despite this, little is known about the regulators and underlying processes involved in the coordination of plant responses to various stressors.

One of the major families of plant TFs is thought to be made up of WRKY proteins. In addition to unique zinc-finger like motifs, members of WRKY family always include one or two WRKY domains, each of which is 60 amino acids in length and identified by the conserved amino acid sequence WRKYGQK at its N-terminus [8, 9]. There are three distinct classes of WRKY TFs based on the presence or absence of certain WRKY domains and the structure of zinc finger motifs [9]. The binding site for WRKY TFs is the W-box (TTGACC/T) found in the promoter region of the respective target genes. The WKRY TFs are essential for plant growth, development, and responsiveness to biotic and abiotic stresses in numerous plant species [10, 11]. Several essential plant functions are either inhibited or repressed by genes belonging to the WRKY family. Previous evidence suggests that several WRKY TFs may coordinately control a single biological process [12, 13]. On the other hand, it is possible that a single WRKY TF regulates many biological activities in plants, even if they seem to be at odds with one another [14].

The WRKY gene family plays a crucial role in the regulation of many abiotic stresses including drought, waterlogging, heat, cold, and salinity [15]. Plant stress responses are controlled by WRKY TFs, which regulate their own expression and interact with other TFs [16]. The *WRKY3* transcription factor is essential for mediating plant defense responses against pathogens. The large proportion of WRKY genes respond to pathogens, elicitors, and defense-related phytohormones like salicylic acid (SA) or jasmonic acid (JA), which indicates the important role of WRKY gene family in plant immunity [17]. The *WRKY3* acts as a positive regulator to defend the plant against necrotrophic fungal infections in *Arabidopsis* [18]. However, integration of WRKY TFs with other TFs in networks that control a wide range of plant biological processes is not well known.

Plant hormones (or phytohormones) naturally occur in plants and play a vital role in several functions, including immunological response, defensive signaling, and growth. Historically, SA, JA, and ethylene (ETH) have been associated with defense responses to pathogen infestation [19]. Nevertheless, plant immunity is not regulated by a single hormone; rather, an intricate network of antagonistic and synergistic interactions involving many plant hormones is involved in the regulation of plant immunity [19]. Pathogens employ numerous strategies to enhance infection by exploiting phytohormone pathways, which demonstrate the importance of phytohormones in plant-pathogen interactions. Pathogens may alter physiological processes like stomatal opening and aging by influencing channels that carry phytohormone signals. In this way, the pathogens infiltrate the plants easily and induce disease symptoms by reducing or blocking the synthesis of phytohormones in their hosts [20, 21]. Pathogen may manipulate phytohormone pathways by directly altering hormone levels, inhibiting their synthesis, or generating hormone mimics [22]. Pathogens may significantly influence the interactions between plants and other pathogens by modifying phytohormone pathways [23]. This alteration has consequences for other processes, including the equilibrium between pathogenesis and mutualism, the manifestation of disease signs, and colonization [23]. The modulation of WRKY transcription factors via phytohormone signaling pathways underscores the intricate regulatory processes involved in plants’ responses to diverse environmental stimuli, such as pathogen infections and stress [12, 24].

Pepper (*Capsicum annuum* L.) is a member of Solanaceae and widely used for culinary and medicinal purposes [25, 26]. The Solanaceae members are very susceptible to bacterial infections, which causes severe production losses. *Ralstonia solanacearum* causes a disastrous disease in pepper known as bacterial wilt. It can adapt to environmental changes and evade the host immune responses by penetrating the root system and multiplying in the xylem tissue [27–29]. Pepper growth and development are significantly impacted by high temperatures and high humidity (HTHH). Although low levels of HTHH may not directly harm pepper plants, they may worsen bacterial wilt disease by fostering the rapid development of *R. solanacearum* and impairing plant immunity [30, 31]. The evolution in pepper may have been impacted by the strong relationship between HTHH and *R. solanacearum* infection. Several earlier studies reported that *CaWRKY6*, *CaWRKY22*, *CaWRKY27* and *CaWRKY40* positively control pepper’s response to combined *R. solanacearum* inoculation (RSI) and HTHH, revealing a tight link between RSI and HTHH [13, 30, 32]. The underlying processes, nevertheless, have not yet been fully analyzed.

This study was aimed at describing *CaWRKY3* (an additional member of the group I WRKY TF family). For this purpose, induction of *CaWRKY3* was observed under *R. solanacearum* infestation and foliar application of SA, MeJA, and ETH. Furthermore, the impact of *CaWRKY3* silencing with virus-induced gene silencing was on pepper immunity was tested. It was hypothesized that foliar application of phytohormones and *R. solanacearum* infestation would increase the transcription of *CaWRKY3.* It was further hypothesized that silencing *CaWRKY3* would result in immunity loss of pepper against *R. solanacearum* infestation. The results would help to understand the role of *CaWRKY3* in pepper immunity against *R. solanacearum* and underlying molecular mechanisms/regulations.

## Results

### Cloning and sequencing analysis of *CaWRKY3*

**Fig. 1:**
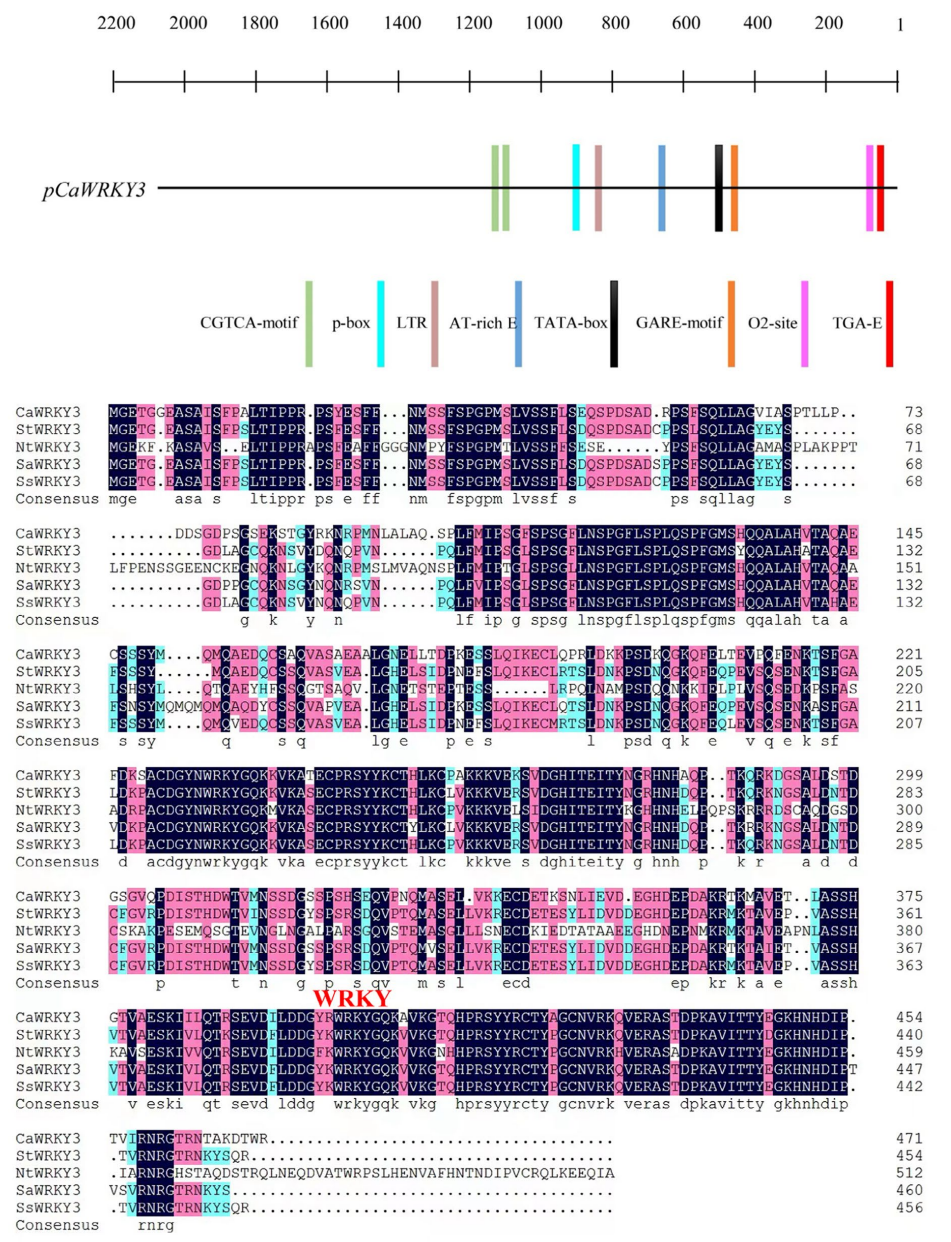
Structural and promoter analysis of *CaWRKY3.* Occurrence of defense related *cis*-elements in promoter region of *CaWRKY3* “A” in the translational start codon ATG is considered as position + 1 (**A**). Multiple alignment of *CaWRKY3* deduced amino acid sequence with proteins from *Solanum tuberosum StWRKY3* (NM_001318664.1), *Solanum acranum SaWRKY3* (KU674829.1), *Nicotiana benthamiana NbWRKY15* (AB711136.1), *Tamarix hispida ThWRKY8* (JX416197.1) and *Diospyros kaki DkWRKY13* (MK737969.1) (**B**) Green shade = 50–75% similarity; red shade = 75–100% similarity, and black shade = 100% similarity. Analysis A and B were assayed by using DNAMAN5

Genomic study (http://passport.pepper.snu.ac.kr) identified a member of the WRKY family (LOC107860501) that has not been studied before in pepper. The *CaWRKY3* was selected for functional characterization and its role in pepper immunity against bacterial pathogen infection, and presence of subset of immunity related *cis* elements, including TGA element, GARE-motif, CGTCA-motif, TATA-box, and W-box in promoter region of *CaWRKY3* indicate its potential role in *Capsicum* immunity (Fig. 1A).

We used gene-specific primers to clone a 1500-base-pair (bp) cDNA fragment encoding the whole open reading frame (ORF) of *CaWRKY3* (Table S1). It was classified as belonging to group I based on its deduced amino acid sequence, which consisted of a total of 500 amino acid residues and had one conserved WRKY domain (Fig. 1A). The size of the anticipated protein is 55 kDa and a projected pI is 7.56. *CaWRKY3* shares 97%, 93%, 82% and 66% amino acid similarity with *StWRKY3*, *SsWRKY3*, *SaWRKY3* and *NtWRKY3* (Fig. 1B).

### Transcriptional expression levels of *CaWRKY3* after *R. solanacearum* inoculation and treatment with different phytohormones

**Fig. 2:**
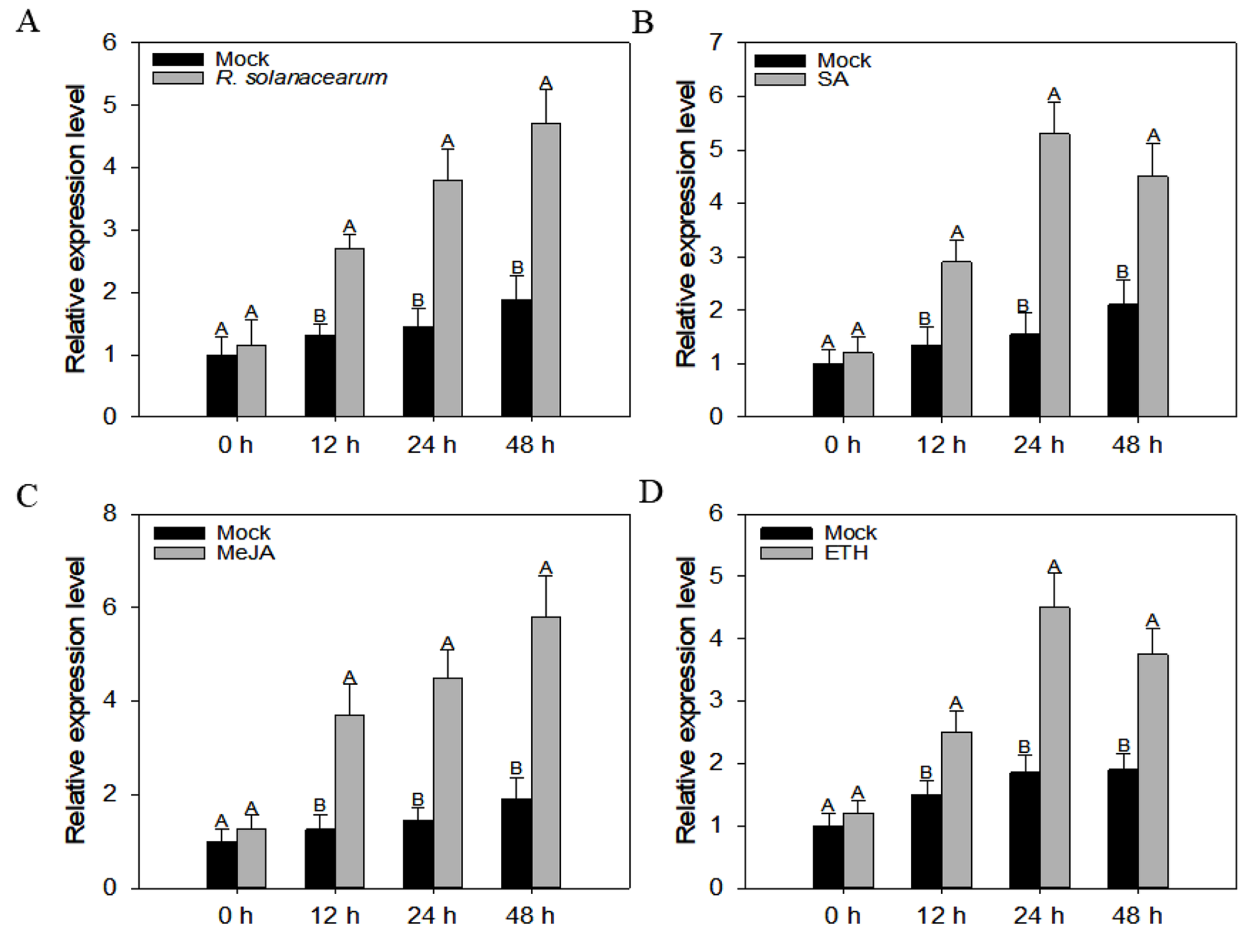
Relative transcriptional levels of *CaWRKY3* in pepper leaves treated with *R. solanacearum* infections and phytohormones quantified by qRT-PCR analysis. Expression levels of *CaWRKY3* in pepper leaves treated with *R. solanacearum* (**A**); foliar spray of 1 mM SA (**B**), foliar spray of 100 μm MeJA (**C**), and foliar spray of 100 μm ETH (**D**). The abundance of RNA synthesis in RSI-treated leaves in comparison to MgCl_2_-control plants (mock) was noted to evaluate the effects of RSI on pepper transcription, where the relative expression level was set to 1. Each treatment consisted of different mock plants. The error bars indicate standard errors of means. Different letters above the bars indicate statistically significant differences between the means of three biological replicates determined by the Fisher protected LSD test

Since promoter region of *CaWRKY3* contains several immune-related *cis* elements, it may have a role in developing immunity against RSI. Compared to mock-treated leaves, leaves exposed to *R. solanacearum* had higher transcriptional expression levels of *CaWRKY3* (Fig. 2A). These elevated transcriptional expression levels of *CaWRKY3* persisted between 0 and 48 h after treatment (hpt). This highest level of expressions was noted at 48 hpt (Fig. 2A).

The response of plants to biotic and abiotic stressors is heavily influenced by signaling pathways controlled by plant hormones such as SA, MeJA, and ETH. The role of phytohormones in the regulation of *CaWRKY3* was investigated by spraying SA, MeJA, and ETH on pepper plants. The data was subsequently put through a quantitative RT-PCR analysis. The qRT-PCR research revealed that the relative transcriptional expression levels of *CaWRKY3* rose from 0 to 48 h following foliar spraying with 1 mM SA in contrast to mock-treated plants. Most transcriptional expressions were still detectable 24 h after treatment (Fig. 2B).

According to qRT-PCR, *CaWRKY3* had higher relative transcriptional expression levels from 0 to 48 h in pepper plants sprayed with 100 µM MeJA in contrast to mock-treated plants. At 48 hpt, top levels of transcriptional expressions were seen (Fig. 2C).

Transcriptional expression levels of *CaWRKY3* in pepper increased from 0 to 48 h following the foliar application of 100 µM ETH, compared to mock-treated plants. There was a peak in this relative transcriptional expression at 24 h after treatment (Fig. 2D).

### *CaWRKY3* silencing by VIGS damaged resistance of peppers to the bacterial pathogen *R. solanacearum* and decreased transcriptional abundance of marker genes linked to plant defence

**Fig. 3:**
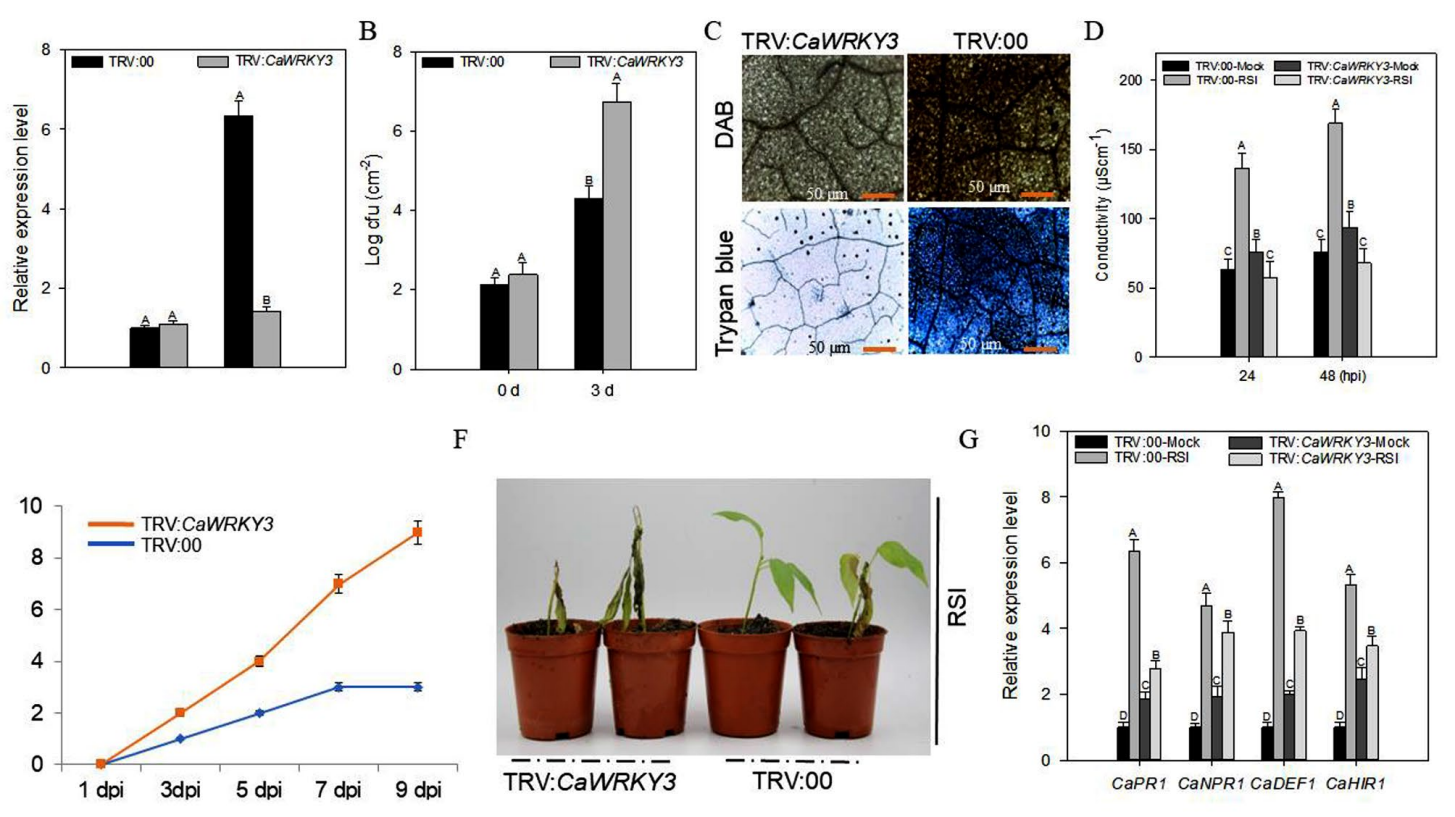
*CaWRKY3*-silencing reduced resistance of pepper to *Ralstonia solanacearum* and down-regulated the immunity-related marker genes. The qRT-PCR analysis of *CaWRKY3* transcripts accumulation in unsilenced (TRV:00) and *CaWRKY3-*silenced plants (TRV:*CaWRKY3*) and *CaWRKY3-*un-silenced plants (TRV:00) (**A**), *R. solanacearum* growth in silenced and un-silenced pepper plants at 0 and 3 days after infection (**B**), DAB and Trypan blue staining in *R. solanacearum*-infected *CaWRKY3-*silenced and *CaWRKY3-*un-silenced leaves (full leaves are provided in Figure S2) (**C**), electrolyte leakage in *CaWRKY3-*silenced and *CaWRKY3-*un-silenced plants (**D**), disease index of silenced and unsilenced plants (**E**), phenotypic response of silenced and unsilenced plants (**F**), qRT-PCR expression of defense-associated marker genes (**G**)

The role of *CaWRKY3* in plant immunity against *R. solanacearum* was studied by silencing of *CaWRKY3* by VIGS. A total 50 *CaWRKY3*-silenced (TRV:*CaWRKY3*) and 50 *CaWRKY3*-unsilenced (TRV:00) plants were used in the VIGS experiment. Six *CaWRKY3*-silenced pepper plants were randomly chosen to assess the efficiency of gene silencing by root infiltration with virulent strain of *R. solanacearum*. Results exhibited that transcriptional abundance of *CaWRKY3* was decreased by ∼ 30% in *R. solanacearum* infected *CaWRKY3*-silenced plants in comparison to *CaWRKY3*-unsilnced plants exhibiting effective silencing of *CaWRKY3* by VIGS (Fig. 3A and B).

The *CaWRKY3*-silenced plants expressed notably higher vulnerability to *Ralstonia* infection, whereas intensity of 3,3′-Diaminobenzidine (DAB) and trypan blue was clearly visible in *CaWRKY3*-silenced pepper plants leaves (Fig. 3C). Electrical conductivity (EC) is marker of ion leakage, and it was measured to check damage of cell membrane and plant cell death after RSI. Results showed that *R. solanacearum* infested *CaWRKY3*-unsilenced plants showed significantly high ion leakage as compared to *R. solanacearum* infested *CaWRKY3*-silenced plants at 48 hpi (Fig. 3D).

Relative disease index assay was carried out to check the intensity of disease levels up to 10 dpi of *R. solanacearum* in *CaWRKY3*-silenced and *CaWRKY3*-unsilenced plants (Table S2). Very strong disease signs were identified in *CaWRKY3*-silenced pepper plants as compared to *CaWRKY3*-unsilnced pepper plants at 10 dpi (Fig. 3E). To check phenotype, 4 *CaWRKY3*-silenced and 4 *CaWRKY3*-unsilenced plants were chosen at random and then treated with *R. solanacearum* in plant roots. Prominent wilting disease symptoms were observed in *CaWRKY3*-silenced pepper plants, whereas very visible disease signs were observed in *CaWRKY3*-unsilnced pepper plants at 10 dpi (Fig. 3F).

Results demonstrated that *R. solanacearum* infected *CaWRKY3*-unsilenced plants showed significantly high ion leakage as compared to *R. solanacearum* challenged *CaWRKY3*-silenced pepper plants at 48 hpi (Fig. 3D). Relative disease index assay was carried out to check the intensity of disease levels up to 10 dpi of *R. solanacearum* in *CaWRKY3*-silenced and *CaWRKY3*-unsilenced pepper plants (Table S2). Very strong disease signs were identified in *CaWRKY3*-silenced pepper plants as compared to *CaWRKY3*-unsilnced pepper plants at 10 dpi (Fig. 3E). To check phenotype, 4 plants of *CaWRKY3*-silenced and 4 plants of *CaWRKY3*-unsilenced were randomly selected and then treated with *R. solanacearum* in plant roots. Prominent wilting disease symptoms were observed in *CaWRKY3*-silenced pepper plants whereas, very feeble disease signs were identified in *CaWRKY3*-unsilnced pepper at 10 dpi (Fig. 3F).

In a quantitative real-time polymerase chain reaction (qRT-PCR) experiment, we looked at the relative expression of many defense-related genes. The transcriptional abundance of multiple defense-related marker genes was reduced in *CaWRKY3*-silenced plants in contrast to *CaWRKY3*-unsilenced plants (Fig. 3G).

### Transient over-expression of *CaWRKY3* instigated cell death resembling HR, synthesis of H_2_O_2_, and transcriptional expression of defense-related marker genes is increased

**Fig. 4:**
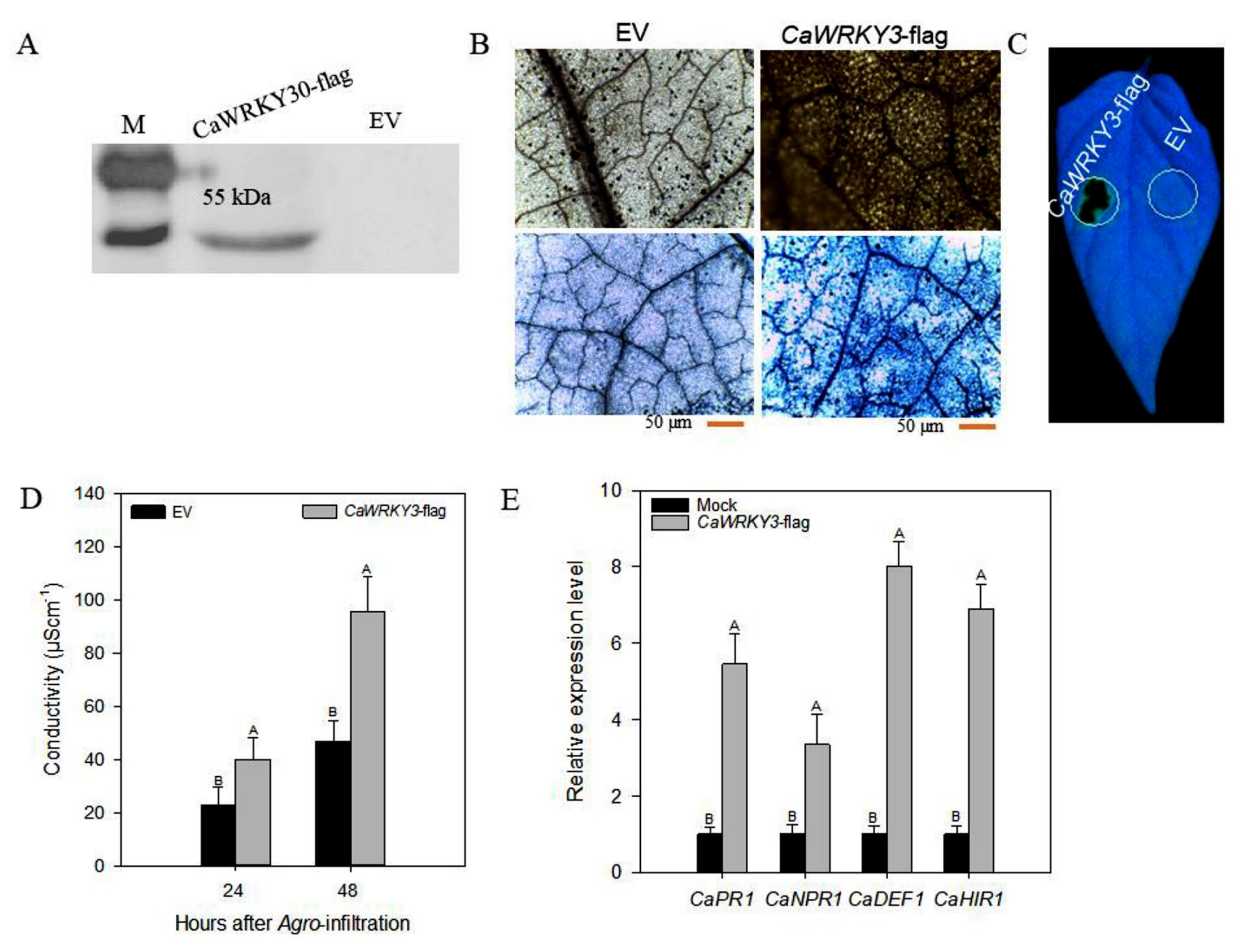
The *CaWRKY3*-transiently over-expressing peppers plants showed HR-like cell death, accumulation of reactive oxygen species and up-regulation of defense-related marker genes. *CaWRKY3* over-expression confirmation by western blot (**A**), HR-like cell detected by phenotype UV light exposure (**B**), HR-like cell detected by DAB and Trypan Blue staining (**C**), ion conductivity to estimate the cell death response in leaf discs (**D**), and qRT–PCR analysis of the transcriptional expression levels of immunity-associated marker genes (**E**)

The *CaWRKY3* has a favorable regulatory role in pepper immunity to *R. solanacearum* inoculation, as indicated by VIGS experiemnt investigating the loss of function of the gene. In order to provide more evidence for this hypothesis, transient over-expression tests of *CaWRKY3* were conducted by injecting healthy pepper leaves with either 35 S:00 (EV) or 35 S:*CaWRKY3*-containing GV3101 Agrobacterium cells. These injections were done to test whether *CaWRKY3* could be overexpressed temporarily. The impact of *CaWRKY3* transient over-expression on HR-like cell death, H_2_O_2_ generation, and transcriptional control of defense-related marker genes was investigated. The results of the Western blot demonstrated that *CaWRKY3* was successfully expressed (Fig. 4A). The experiments with trypan blue and DAB staining verified the formation of H_2_O_2_ and the presence of HR-like cell death in the plant leaves. Following the temporary overexpression of *CaWRKY3*, DAB staining in dark brown and extensive trypan blue were seen in plant leaves. These stains served as indications of HR-like cell death and H_2_O_2_ production (Fig. 4B and C). Electrical conductivity experiment was carried out to check the intensity of plasma membrane damage induced by transient over-expression of *CaWRKY3.* Our data exhibited that electrolyte leakage was significantly high in pepper leaves transiently over-expressing *CaWRKY3* as compared to pepper leaves transiently over-expressing empty vector at 24 and 48 h of Agro-infiltration (Fig. 4D and E).

### Relationship between *CaWRY3*, *CaWRKY6*, *CaWRKY22*, *CaWRKY27*, and *CaWRKY40*

**Fig. 5:**
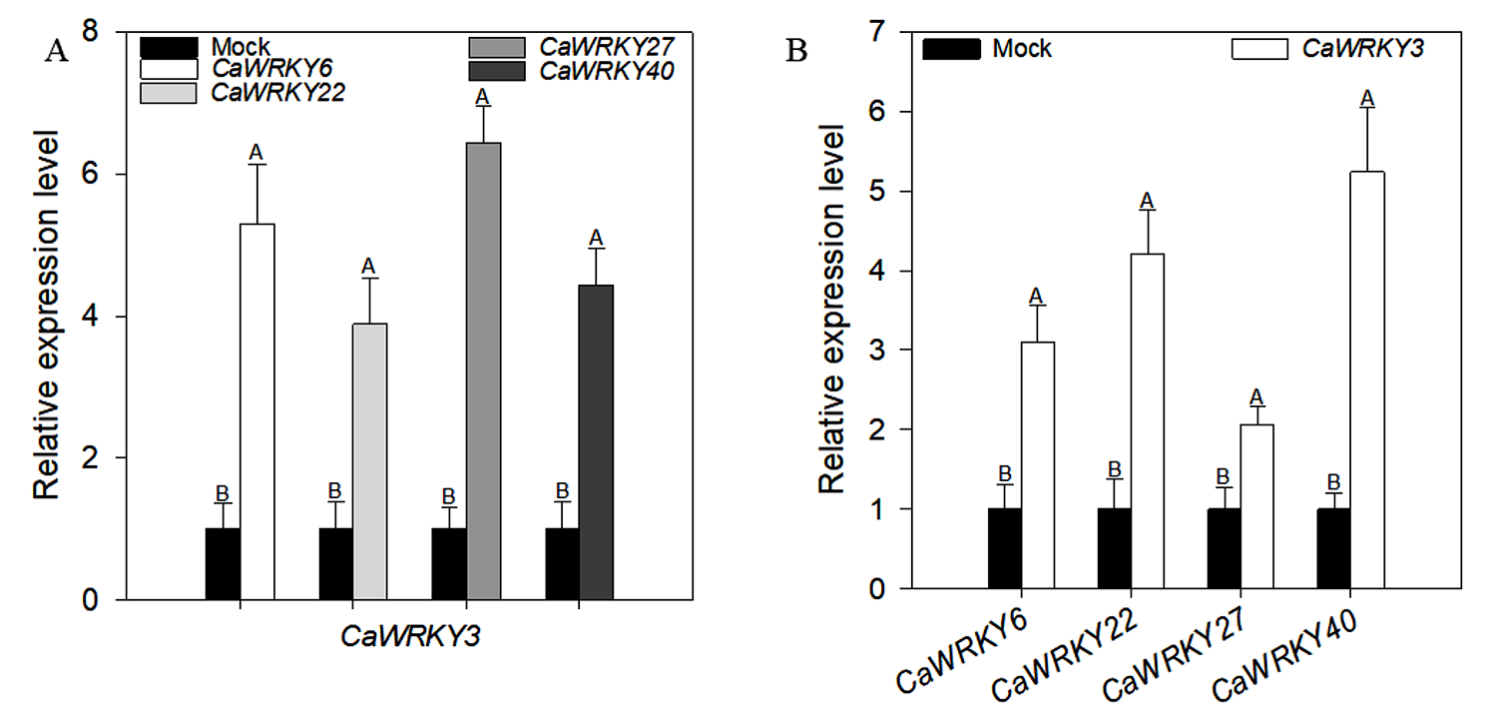
Relationship between *CaWRKY3*, *CaWRKY6*, *CaWRKY22*, *CaWRKY27*, and *CaWRKY40*. Transcriptional expression of *CaWRKY3* in pepper leaves transiently over expressing *CaWRKY6, CaWRKY27 and CaWRKY40* (**A**), and transcriptional expression of *CaWRKY6, CaWRKY27 and CaWRKY40* in pepper leaves transiently over-expressing *CaWRKY3* (**B**)

Previous studies have suggested that *CaWRKY40* was expressionally and functionally associated to various WRKY TFs, including *CaWRKY6* and *CaWRKY22*. We also found previously that various WRKY TFs were expressionally and functionally interconnected. This notion indicates that *CaWRKY3* might also be related to various WRKY TFs responsible for pepper immunity to RSI (Fig. 5A and B).

### *CaWRKY3*-OX (Over-expression) increased the resistance of transgenic *Arabidopsis* plants to bacterial infection and up-regulated the levels of immunity-related marker genes transcription

**Fig. 6:**
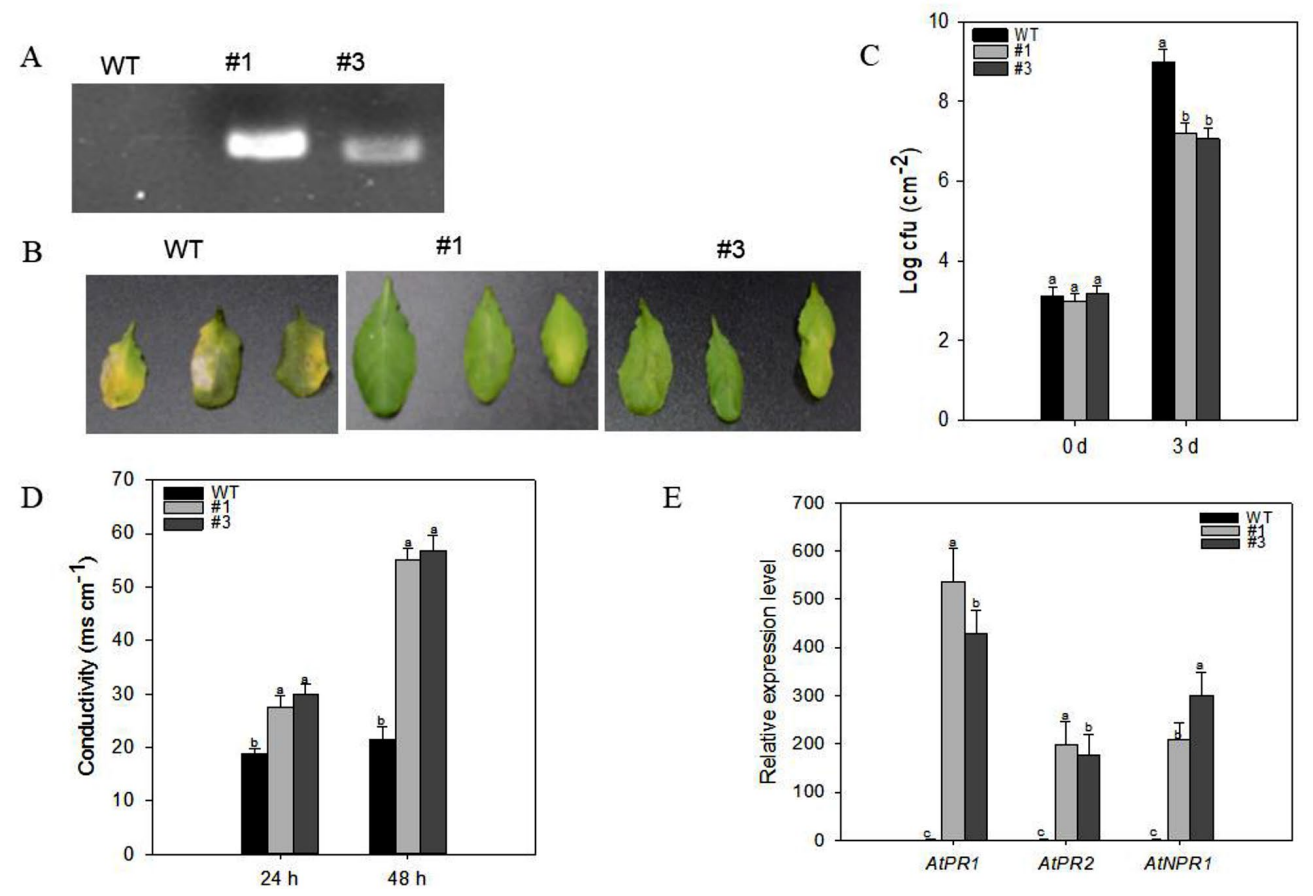
*CaWRKY3* over-expression in transgenic *Arabidopsis* plants enhanced the resistance against *Pseudomonas syringae* pv tomato (*Pst*) DC3000 inoculation. *CaWRKY3* gene amplification by qRT-PCR (**A**), wild type and *CaWRKY3*-OX strains of *Arabidopsis* (**B**), disease index in wild type and *CaWRKY3*-OX strains of *Arabidopsis* (**C**), ion conductivity to estimate the cell death response in leaf discs (**D**), and transcriptional expression of SA-dependent PR genes involved in immunity (**E**)

Production of *CaWRKY3* transgenic plants in *Arabidopsis* and over-expressing the complete precursor under the control of the 35 S promoter allowed researchers to learn more about the role of *CaWRKY3* in plant defense in vivo. This was made feasible by the extreme difficulty of maintaining metamorphosis in pepper plants. The goal was to learn *CaWRKY3* functions in real-life plant defense. The genomic DNA was isolated and analyzed from *CaWRKY3*-OX transgenic *Arabidopsis* plants and wild plants using PCR. This was done in a variety of homozygous T3 *Arabidopsis* lines to verify that *CaWRKY3*-OX transgenic expression was successful (Col-0). Only two *CaWRKY3*-OX plants had their *CaWRKY3* gene amplified by PCR; however, none of the wild-type *Arabidopsis* plants amplified the gene (Fig. 6A). In typical growing circumstances, *CaWRKY3*-OX *Arabidopsis* plants showed no observable phenotypic differences from wild-type plants. Needle-free syringe injections of *P. syringe* pv *tomato* DC3000 were used to test the wild type and *CaWRKY3*-OX strains of *Arabidopsis* for resistance to the bacterial pathogen. Standard chlorotic signals were found on infected leaves of wild type plants three days after inoculation, but not on infected leaves of *CaWRKY3*-OX plants (Fig. 6B).

To determine whether disease symptoms were caused by bacterial multiplication in plants, researchers looked at the growth of the bacterial pathogen in plant leaves at 0 and 3 days after the first infection. In comparison to *CaWRKY3*-OX plants, wild type plants produced bacterial pathogens more rapidly (as measured by higher cfu levels at time intervals of 0 and 3 days). These findings were apprehended via a comparison of the two plant types (Fig. 6C). Increased plant resistance to bacterial infection may be attributable to higher *CaWRKY3* expression levels, as shown by our findings. To monitor plasma membrane damage and cell mortality due to the HR reaction, an electrolyte leakage experiment was performed. At 0, 12, and 24 h after infection with *P. syringe* pv *tomato Pst* DC3000, compared to wild type plants, the *CaWRKY3*-OX leaves showed higher amounts of electrolyte ion leakage (Fig. 6D).

We compared the *CaWRKY3*-OX transgenic *Arabidopsis* plants to the wild type plants in terms of the transcriptional expression of SA-dependent PR genes involved in immunity using qRT-PCR. The *CaWRKY3*-OX plants showed greater transcriptional abundance of immunity-related marker genes compared to wild type plants 48 h after being treated with *P. syringe* pv *tomato Pst* DC3000. This was true irrespective of the genotypes of the plants (Fig. 6E). The enhanced disease resistance of *CaWRKY3*-OX against bacterial pathogen infection may be due to SA-dependent immunological mechanism, as shown by the qRT-PCR experiment.

## Discussion

The WRKY TF family is abundant in plant cells. The members of this family have been demonstrated to play important roles in the control of plant immunity in *Arabidopsis* and rice. Previous research has shown that WRKY proteins found in a wide range of plant species have high degrees of structural similarity but vastly different functional properties [33, 34]. However, the function of WRKY TFs in immunological responses of non-model plants, such as pepper, has received less attention. The *CaWRKY3* (a member of WRKY TF family group I) was the focus of our investigation as we defined its functional properties. According to our findings, *CaWRKY3* functions as a positive regulator of pepper immunity to RSI together with *CaWRKY6*, *CaWRKY22*, *CaWRKY27*, and *CaWRKY40*, which are also members of the WRKY transcriptional web.

The promoter region of *CaWRKY3* contains immunity-sensitive cis-elements such as the CGTCA-motif, p-box, TATA box, and W-box, lending credence to the hypothesis that it plays a role in regulating pepper immunity. Further supporting the role of *CaWRKY3* in pepper immunity are the results of qRT-PCR reaction, which demonstrated that *CaWRKY3* transcriptional levels were up-regulated with *R. solanacerum* infection. Genes are known to have a part in the body’s response to stress if their expression level rises in such circumstance [27]. We hypothesized that *CaWRKY3* would regulate pepper immunity in a manner that would make the plant more resistant to *R. solanacearum*. Results of the VIGS experiment for *CaWRKY3* silencing, transient over-expression assay for gain-of-function analysis, and persistent transgenic overexpression of *CaWRKY3* in *Arabidopsis* all supported our hypothesis.

The loss of function of *CaWRKY3* by VIGS assay increased the vulnerability of pepper plants to RSI. This decreased immunity was accompanied by increase in growth of inoculated bacterial pathogen *R. solanacearum* and down regulation of HR-related *CaHIR1* [35] pathogenesis-related *CaPR1* [36] and SA-related *CaNPR1* [37] and JA-related *DEF1* [38]. On the other hand, pepper plants transiently over-expressing *CaWRKY3* showed cell death resembling HR, H_2_O_2_ synthesis, and up-regulation of immunity related marker genes, including *CaHIR1*, *CaNPR1, CaPR1*, and *CaDEF1*. These results of *CaWRKY3*-silencing and gain-of-function experiments significantly suggest that *CaWRKY3* functions as a positive regulator of pepper’s immunity and HR-like cell death against bacterial pathogen infection.

Furthermore, transgenic *Arabidopsis CaWRKY3*-OX plants exhibited higher resistance compared to plants of the wild type. This data suggested that *CaWRKY3* positively regulate pepper plant immunity and play a critical part in the defense of plants against bacterial pathogen. Previous studies exhibited that *WRKY3* is a positive regulator of plant defense against *F. oxysporum* in *Lilium regale* and transcriptional levels of JA-biosynthesis, SA-signal transduction-related marker genes were up-regulated in *LrWRKY3* transgenic tobacco lines [33, 34]. It can be suggested that *CaWRKY3* is up-regulated upon inoculation of *R. solanacearum*.

The *CaWRKY3* was constantly detected to be triggered by foliar spraying of phytohormones including SA, JA, and ETH. The SA, JA and ETH-dependent defense related marker genes such as *CaPR1* [36], *CaNPR1* [37], *CaDEF1* [38] and *CaHIR1* [35] were down-regulated upon silencing of *CaWRKY3*, while these all defense associated marker genes were up-regulated in *CaWRKY3*-transiently over-expressing pepper plants showing that *CaWRKY3* is involved in SA, JeA and ETH synergistically mediated defense signaling, hence leads to PTI.

Genome-wide studies showed the involvement of many WRKY TFs in plant immunity [12, 39]. By functional genomics studies *WRKY11* [40], *WRKY17* [41], *WRKY18* [42], *WRKY22* [13], *WRKY25* [43], *WRKY28* [44], *WRKY33* [45], *WRKY38* [46], *WRKY45* [47], *WRKY46* [48], *WRKY53* [49], *WRKY6*2 [50], *WRKY70* [51], and *WRKY75* [52] have been identified as having functional characteristics in *Arabidopsis*’ immunity, these were either positive regulator or negative regulator of plant immunity. These WRKY TFs have been recommended to assimilate into a transcriptional network consisted of + ve and –ve feedback loops and feed forward modules [16].

However, the formulation of these WRKY TFs networks in various plants species is poorly understood. Our previous experiments data exhibited that *CaWRKY6*, *CaWRKY22*, *CaWRKY27*, *CaWRKY30* and *CaWRKY40* are positive regulator of pepper’s immunity against RSI [11, 32, 53–55], *CaWRKY58* was found to be negative regulator in pepper’s resistance to RSI.

## Conclusions

The *CaWRKY3* was functionally characterized in the current study. The findings indicated that *CaWRKY3* functions as a positive regulator of pepper immunity to RSI together with *CaWRKY6*, *CaWRKY22*, *CaWRKY27*, and *CaWRKY40*, which are also members of the WRKY transcriptional web. An increase in *CaWRKY3* transcription was seen after temporary over-expression of previously identified *CaWRKY6*, *CaWRKY22*, *CaWRKY27*, and *CaWRKY40*, and the same was observed after transient over-expression of *CaWRKY3*, showing the existence of WRKY TF networks and positive feedback.

## Methods

### Plant material and growth circumstances

We procured pepper (‘Mexi’) and tobacco (*Nicotiana tabacum* L.) seeds from the Ayub Agriculture Research Institute (AARI), Faisalabad, Pakistan. Soil composed of peat, moss, and perlite [2/1(v/v)] was used to plant the seeds in plastic containers.

### Vectors construction

The gateway cloning method was used for vector creation. To generate vectors for VIGS, we chose a 288 bp fragment from the 3’-untranslated region (UTR) of *CaWRKY3* and validated its specificity by BLASTing it against the genome sequences of CM334 (http://peppergenome.snu.ac.kr/).

### Pathogens and inoculation procedures

Plants infected with *R. solanacearum* in South Punjab produced a strain of the bacterium that is very pathogenic (Pakistan). Tetrazolium chloride was used to purify the exudates from the stem and stem vascular tissue of these diseased plants. Sucrose, peptone, and agar (SPA) medium was used to cultivate a highly infectious *R. solancearum* strain overnight at 200 rpm and 28 °C in a temperature-control shaker (200 g potatoes, 20 g sucrose, 3 g beef extract, 5 g tryptone, and 1 L of double-distilled ddH_2_O). *R. solanacearum* culture was centrifuged for 10 min at 6500 rpm and 28 °C. After discarding the supernatant, the pellet was dissolved in sterile, distilled 10 mM MgCl_2_. When using bacteria, a concentration of 0.8 log (108 cfu mL^-1^) was used (at an optical density of 600 nm, or OD600). To investigate how RSI affects *CaWRKY3* transcriptions levels and pepper plants’ resistance to RSI, a needleless syringe was used to inject 10 ml of *R. solanacearum* into the upper third of the leaves of pepper plants. At certain periods, leaves from treated plants were collected for DAB and trypan blue staining and RNA extraction. *CaWRKY3* knocked-down pepper plants were subjected to root injury (through glass road cuts) and *R. solanacearum* infiltration to evaluate phenotypic changes after RSI. The plants infected with *R. solanacearum* were grown in a greenhouse at a temperature of 28 degrees Celsius, with a light intensity of 60 to 70 micromoles per square meter per second, a humidity level of 70%, and a photoperiod of 16 h of light and 8 h of darkness. The potentially lethal pathogen *Pseudomonas syringae* pv. Tomato DC3000 (*Pst* Dc3000) was also used. Overnight, the bacterial pathogen was cultivated at 200 rpm and 28 °C in SPA medium supplemented with the necessary antibiotics for treating *Arabidopsis* plants. This pathogen culture solution was centrifuged for 10 min at 28 °C and 200 rpm. The pellet was centrifuged and then suspended at a concentration of 10^5^ cfu mL^-1^ in distilled water with 10 mM MgCl_2_. A needleless syringe was used to inject *Pst* Dc3000 into the leaves of an *Arabidopsis* plant that was five to six weeks old. After 18 h in the humid chamber, the pathogen-treated *Arabidopsis* plants were transferred to the growth room. Phenotyping, electrolyte conductivity testing, and RNA isolation all required periodic sample collection.

### Foliar application of phytohormones

Pepper plants that were in the 4-leaf stage were sprayed with 1 mM salicylic acid (SA), 100 µM methyl jasmonate (MeJA), and 100 µM ethylene (ETH) to investigate the function of these phytohormones. The analytical grade SA, MeJA and ETH were obtained from Merck. Sterile solution of ddH_2_O was used to treat the mock plants. At various time periods, leaf samples of pepper plants treated with phytohormones, and mock plants were collected for subsequent experiments.

### VIGS (virus-induced gene silencing) experiment

A VIGS approach based on the Tobacco Rattle Virus (TRV) was applied to silence *CaWRKY3* in pepper. *A. tumefaciens* strain ‘GV3101’ with PYL192 and PYL279- or PYL279 was grown over night in a thermocontrol shaker at 200 rpm and 28 °C. After that, cultured *A. tumefaciens* was centrifuged for 10 min at 6800 rpm and 28 °C. The liquid supernatant was discarded, and the solid pellet at the bottom was dissolved in induction media [10 mM MES, 10 mM MgCl_2_, 200 M acetosyringone, (pH 5.6)] and diluted to an optical density of 0.6 (OD600). *A. tumefaciens* strains containing PYL192 and PYL279, PYL 279-*CaWRKY3*, and PYL192-PDS were respectively combined in a ratio of 1:1. A syringe without a needle was used to inject this solution into the cotyledons of pepper seedlings that are two weeks old. After that, treated pepper plants were placed in a dark at 16 °C in the growth room. with 45% relative humidity for 56 h. After 56 h, the plants were transferred to the growth chamber, where they were subjected to a 16-hour light/8-hour dark cycle, 25 ± 2 °C temperatures, 70% relative humidity and 60–70 mol photons m^-2^s^-1^ light intensity.

### CaWRKY3 transient over-expression assay

*A. tumefaciens* strain ‘GV3101’ was grown overnight to an OD600 of 1 on LB medium bearing necessary antibiotics in a temperature-control shaker at 28 °C and 200 rpm. It included 35 S: CaWRKY3-flag and 35 S:00 (empty vector). After centrifuging the culture of *A. tumefaciens*, the solid pellet was dissolved in induction media removed [10 mM MES, 10 mM MgCl2, 200 M acetosyringone, (pH 5.6)] after the supernatant liquid was removed and adjusted to an optical density (OD) value of 0.8. A needleless syringe was used to inject this into the vigorous pepper plants’ leaves. Plants were then placed in a growing chamber and samples were taken at different intervals to be used in further trials.

### Histochemical staining

The leaves of *CaWRKY3*-transiently over-expressed plants, plants with and without *CaWRKY3* silencing were observed, as well as leaves that were infected with *R. solanacearum* were histochemically stained using trypan blue and 3-diaminobenzidine (DAB) staining. After treating the pepper leaves, they were placed in a solution of chloral hydrate (2.5 g of chloral hydrated dissolved in 1 mL of distilled water) after being heated in a solution of trypan blue staining (10 mL lactic acid, 10 mL glycerol, 10 mL phenol, 40 mL ethanol, and 10 mL ddH_2_O) for 30 min. The leaves were re-boiled three times for 20 min each to remove the remaining discoloration. Finally, samples were stored in glycerol at a 70% concentration. By soaking treated pepper leaves in a 1 mg/ml DAB solution overnight at room temperature, the DAB staining test was performed. Absolute ethanol, glycerol, and lactic acid at a ratio of 1:1:3 (vol/vol/vol) solution was used to remove the DAB stain from the pepper leaves, and the leaves were then preserved in a 95% absolute ethanol solution. Photos of dyed leaves with trypan blue, and diaminobenzidine were taken by microscope (Leica, Wetzlar, Germany).

### RNA isolation and real-time qRT-PCR

The TRIzol reagent technique was used to extract the total RNA from the pepper leaves (Invitrogen, Carlsbad, California, USA), and it was then subjected to reverse transcription using a Prime Script RT-PCR Kit (TAKARA, Dalian, China). Quantitative real-time polymerase chain reaction (qRT-PCR) was used to investigate the relative transcriptional levels of certain marker genes. This was accomplished by utilizing a SYBR premix Ex Taq II system (TAKARA Perfect Real Time) in conjunction with a Bio-Rad Real time PCR system (Bio-Rad, Foster City, California, United States). Real-time quantitative PCR and data processing were performed in the same manner as described earlier [13]. The primers used in qRT-PCR are given in Table S3. The original gel images are given in Fig. S1.

### Ion Conductivity

We examined electrical conductivity (ion leakage) in previously described manner with some minor adjustments [11]. Six discs of leaf tissue using a hole-puncher, the 4 mm-diameter pieces were taken, incubated in 10 ml of ddH_2_O after being sterilized by washing them three times in ddH_2_O. These discs were incubated for 1 h at room temperature while being gently shaken at 60 rpm. Conductivity meter was used to measure electrical conductivity (Zurich, Switzerland: Mettler Toledo 326).

### Immunoblotting

Using a protein extraction buffer, the whole protein in pepper leaves was extracted, as reported elsewhere [29, 56]. After extraction, the protein was left to react with anti-HA agarose at a temperature of 4 °C for overnight (Thermo Fisher Scientific; Waltham, Massachusetts; United States). Using a magnetic crack, beads were collected. They were then washed three times in tris-buffer saline (TBS) and tween 20 (0.05%). Anti-HA peroxidase (Abcam, Cambridge, UK) and immunoblotting were both employed to investigate the eluted protein.

### Transgenic Arabidopsis plants that overexpress *CaWRKY3*-OX

Using the BP method, *CaWRKY3*’s full-length cDNA was cloned into the vector pDONR207. This construct was then ligated into the PK7WG2 target vector. Then, the vector PK7WG2 bearing the CaWRKY3-35 S promoter of the cauliflower mosaic virus was introduced into the ‘GV3101’ strain of *Agrobacterium tumefaciens*. By using the floral dip approach, transgenic *Arabidopsis* plants that overexpress *CaWRKY3*-OX were created using an agrobacterium-mediated transformation process [57, 58]. To create separate transgenic lines, seeds from these *CaWRKY3*-OX over-expressing transgenic *Arabidopsis* seedlings were collected and seeded in Petri plates using MS agar medium containing 50 g ML^-1^ kanamycin. PCR was used to verify that the *CaWRKY3* cDNA was successfully inserted into the transgenic Arabidopsis plants’ genome.

### Electronic supplementary material

Below is the link to the electronic supplementary material.

Supplementary Material 1

Supplementary Material 2

## Data Availability

All data are within the manuscript and supplementary files.
