# Supplementary material for: Pepper immunity against Ralstonia solanacearum is positively regulated by CaWRKY3 through modulation of different WRKY transcription factors
Source: BMC Plant Biol. 2024 Jun 10;24:522. doi: 10.1186/s12870-024-05143-z (PMC11163704; doi:10.1186/s12870-024-05143-z)
Supplement: Supplementary file 1 — Supplementary Material 1 [file 12870_2024_5143_MOESM1_ESM.docx]

Pepper immunity against *Ralstonia solanacearum* is positively regulated by *CaWRKY3* through modulation of different WRKY Transcription Factors

**SUPPLEMENTARY MATERIALS**

**Table S1. Pepper primers used for vectors’ construction**

| **Gene** | **Forward primer (5’-3’)** | **Reverse primer (3’-5’)** | **Size (bp** |
| --- | --- | --- | --- |
| ***CaWRKY3^1^*** | ATGGGAGAAACCGGGGGAGAA | TCAACGCCAAGTATCTTTGGC | 1500 |
| ***CaWRKY30^2^*** | GAGAAGCTTCAGCAATATC | TGAAACCAGAGGGAATCATG | 311 |
|  |  |  |  |

**^1^Primers for full length cloning of *CaWRKY3***

**^2^Primers for construction of TRV:*CaWRKY3* vector**

**Table S2. Disease index for *Ralstonia* infected pepper plants**

| **score** | **Condition level** |
| --- | --- |
| 0 | Capsicum plants are normal and asymptomatic at 0. |
| 1 | Plants with slight withering, the basal 1-2 leaves withered, the apical region of plant is normal. |
| 2 | Leaves in addition to the top leaves, 1: 2 leaves withered, the apical region of plant is normal. |
| 3 | 2/3 of the leaves of pepper plant withered while top pepper plant is normal. |
| 4 | Most of the leaves were withered including the apical leaves |
| 5 | Whole plant was withered or dead. |
|  |  |

**Table S3. Primers used in Real time RT PCR analyses.**

| **Gene** | **Accession number** | **Forward primers (5’-3’)** | **Reverse primers (3’-5’)** | **Size (bp)** |
| --- | --- | --- | --- | --- |
| ***CaWRKY30^1^*** | LOC107860501 | AGGTCCAATGTCCTTAG | CATCGGTCGATTCTTCC | 178 |
| ***CaPR1*** | AF348141.1 | GCCGTGAAGATGTGGGTCAATGA | TGAGTTACGCCAGACTACCTGAGTA | 108 |
| ***CaNPR1*** | X61679.1 | ACTTCTTCGCCGACGCCAAG | GCCAACACATTCACCAGAGCATC | 190 |
| ***CaDEF1***  ***CaHIR1*** | AF442388  AAX20040 | GTGAGGAAGAAGTTTGAAAGAAAGTAC  CCTGCAATGTTTGCTCATTTGAC | TGCACAGCACTATCATTGCATACAATTC  CATGGGAATCGTTGATCTTAATC | 267  164 |
| ***CaActin*** | GQ339766 | AGGGATGGGTCAAAAGGATGC | GAGACAACACCGCCTGAATAGC | 225 |
| ***18srRNA*** | EF564281 | CCGGTCCGCCTATGGTGTGCACCGGTCGTC | GCAGTTGTTCGTCTTTCATAAATCCAAGAA | 285 |
| ***CaWRKY6***  ***CaWRKY22*** | KF736800  [CA08g07730](http://peppergenome.snu.ac.kr/gprotein.php?a=dv&id=370480257) | GGTAGCTAGACAATTATGCTGC  GAGGCTGCACAGCTAGTTCCA C | CAAAAAAAAATCTTATCAACTTG  CACCAAGAACAGAGAGGGG | 142  162 |
| ***CaWRKY27*** | DQ102364.1 | CTGAGCAAGATGATTCCGAGAA | ATTGGCACTGACACCACTCT | 148 |
| ***CaWRKY40*** | AAX20040.1 | AAGTCCAGCAGAGCAGTCAA | AACAATTGTCTAAGCCATCCG | 152 |

**^1^Specific primers to detect relative expression of *CaWRKY3* designed according to the sequence in 3’UTR**

**Specific primers for marker genes**
