# Supplementary material for: Pepper immunity against Ralstonia solanacearum is positively regulated by CaWRKY3 through modulation of different WRKY transcription factors
Source: BMC Plant Biol. 2024 Jun 10;24:522. doi: 10.1186/s12870-024-05143-z (PMC11163704; doi:10.1186/s12870-024-05143-z)
Supplement: Supplementary file 2 — Supplementary Material 2 [file 12870_2024_5143_MOESM2_ESM.docx]

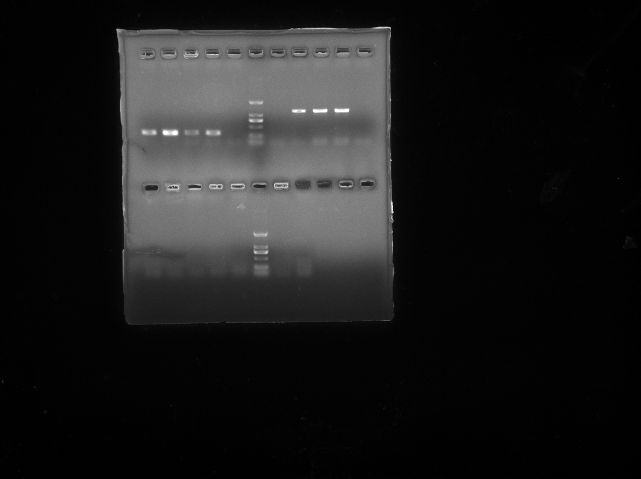


Figure S1. Raw gel image used to determine the overexpression of *CaWRKY3*

| Unsilenced | 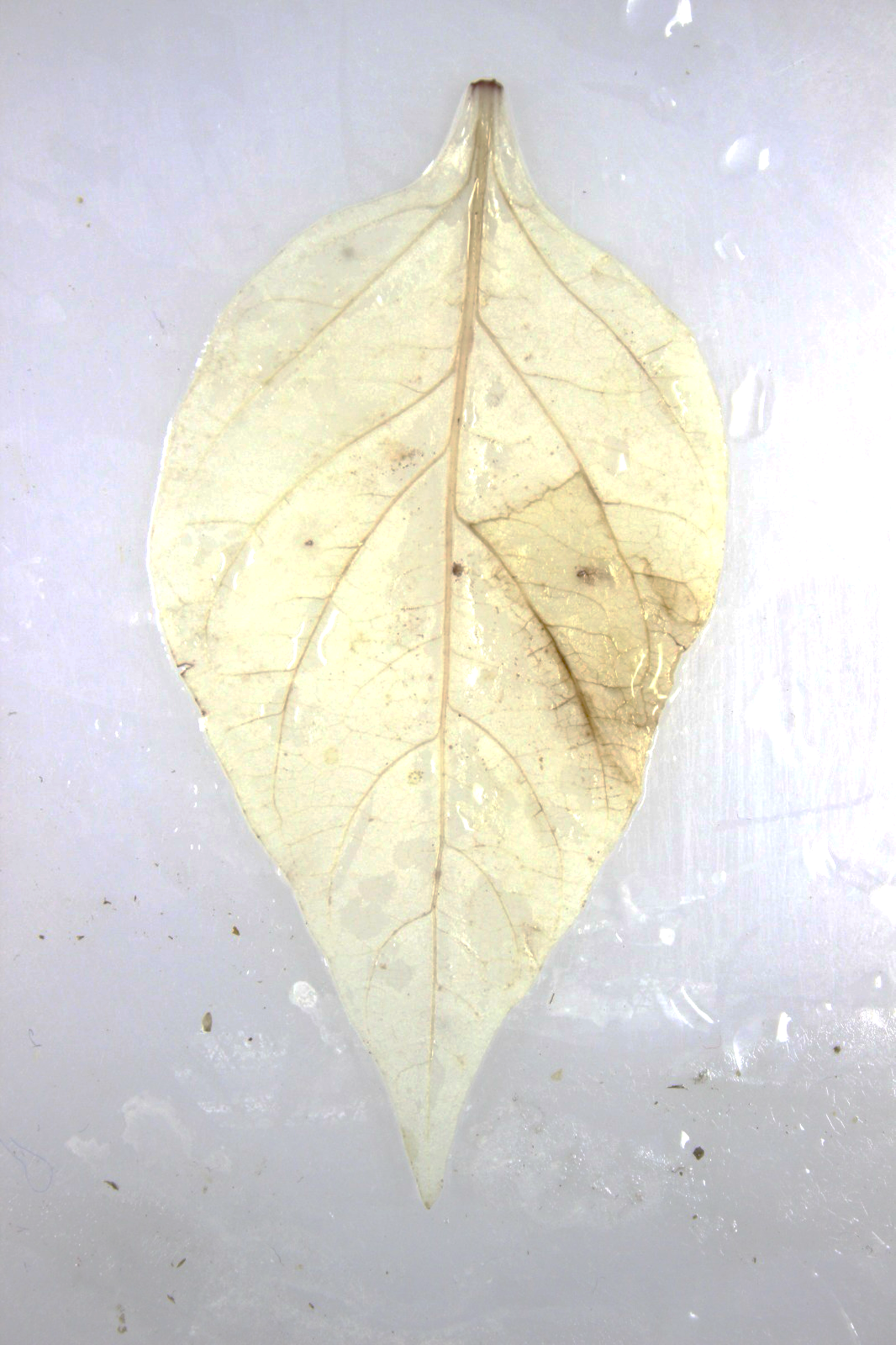 | 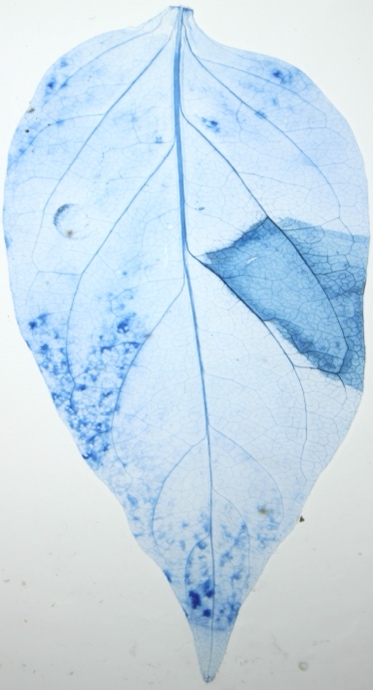 |
| --- | --- | --- |
| Silenced | 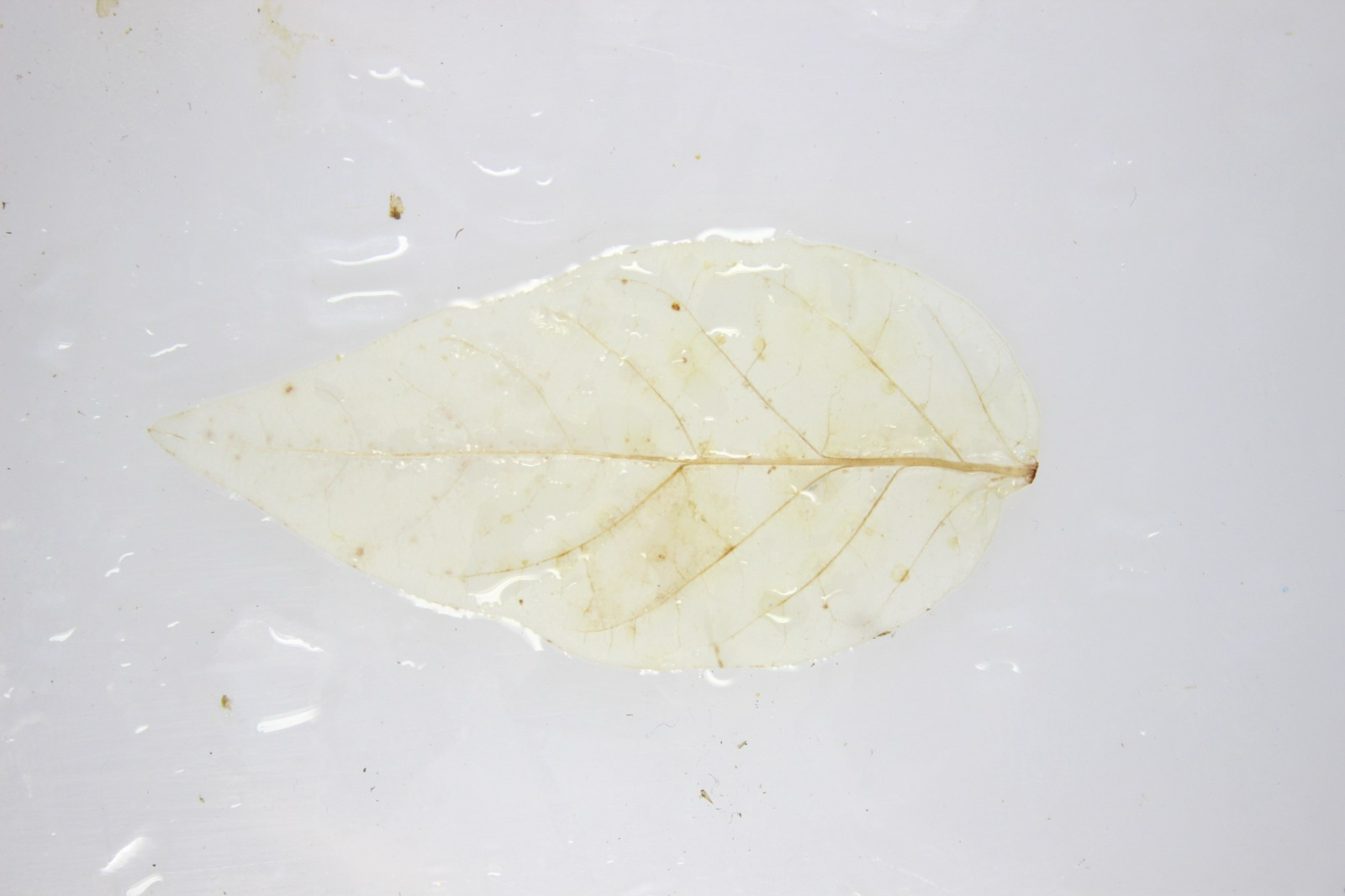 | 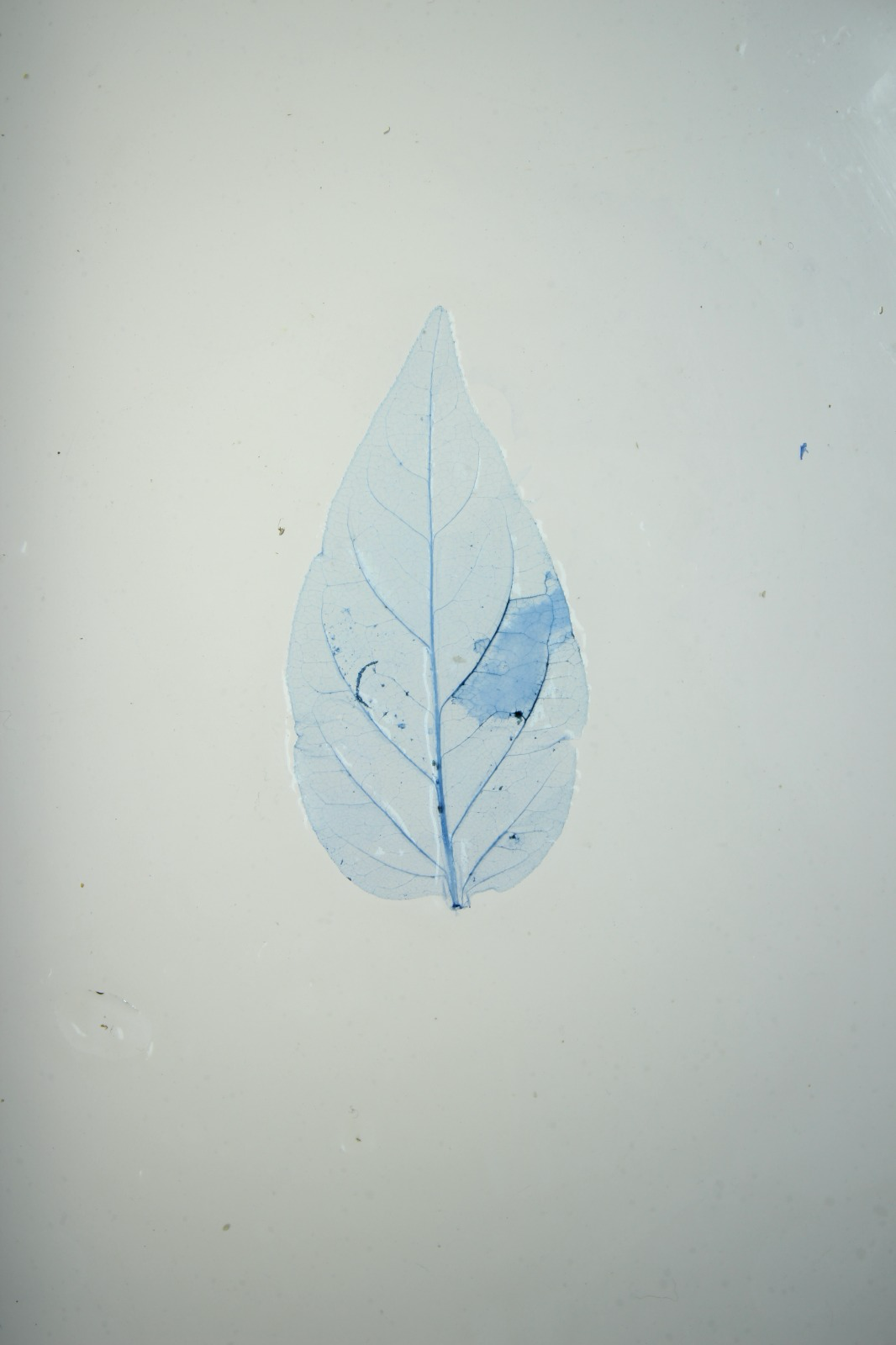 |
|  | DAB Staining | Trypan blue staining |

Figure S2. Leaf images of DAB and Trypan blue staining in *R. solanacearum*-infected *CaWRKY3-*silenced and *CaWRKY3-*un-silenced leaves
